# Socio-Cultural Factors Delaying Treatment in a Patient with Late-Onset Schizophrenia

**DOI:** 10.1155/2022/9689732

**Published:** 2022-02-26

**Authors:** Ho Teck Tan, Manu Lal

**Affiliations:** Institute of Mental Health, Singapore

## Abstract

Up to 23.5% of patients with schizophrenia have onset of illness after the age of 40. We report a case of a 57-year-old lady who had been sitting continuously on the toilet for 2.5 years because of persecutory delusions and somatic passivity symptoms. She was diagnosed with late-onset schizophrenia and her symptoms improved with risperidone. In this case report, we describe the phenomenology of her psychotic symptoms and explore the socio-cultural factors behind the long duration of untreated psychosis (DUP). We conclude that more can be done to improve mental health awareness and reduce the social stigma associated with mental illness.

## 1. Introduction

Bleuler [1] first described late-onset schizophrenia in 1943, where the onset of illness occurred between 40–60 years of age. The revised edition of DSM-III included the diagnosis of late-onset schizophrenia, even though the age criterion was omitted in subsequent editions of the DSM [2]. Late-onset schizophrenia has a higher predominance in women [3]. A study of the phenomenology of late-onset schizophrenia found the 3 most common phenomenon to be persecutory delusions (83%), delusion of influence, and hallucinations (both 66%) [4]. Various studies showed that 48%–61% of patients with late-onset schizophrenia experienced full remission of their symptoms with treatment [5]. In Singapore, the median duration of untreated psychosis (DUP) in patients with 1^st^ episode psychosis is 12 months [6]. In this case report, we describe the phenomenology of a 57-year-old female patient with late-onset schizophrenia who had been sitting continuously on the toilet for 2.5 years and explore socio-cultural factors behind the long duration of untreated psychosis (DUP).

## 2. Case Presentation

### 2.1. Previous History

A 57-year-old woman was admitted to a tertiary psychiatric hospital in Singapore after being brought by ambulance. She had been sitting continuously on the toilet for 2.5 years before being sent to hospital to receive treatment.

She was the second youngest out of the 12 siblings from an ethnic Chinese family in Malaysia. She studied up to primary 5 and had stopped school due to financial constraints at home. She did odd jobs before coming to Singapore to work as a seamstress when she was 23 years old. She was married at the age of 24 and became a housewife.

She had stayed with her husband and her 27-year-old journalist son in a 3-room flat. She had a close relationship with her husband who was her sole caregiver, while her son was largely uninvolved in her care. She had a limited social circle and only maintained contact with one of her siblings via telephone.

She had no known family history of mental illness and had no significant medical illness. Prior to her illness she was described as an extroverted and easy-going person who enjoyed shopping and making new friends. She was a law-abiding citizen who did not engage in any illicit substance use, smoke, or drink alcohol.

She first experienced a strange sensation of a strong force holding her shoulders down while sitting on the toilet when she was 47 years old. She began spending long hours in the toilet as she constantly felt the sensation of incomplete voiding in her abdomen even after defecation. She had visited the general practitioner twice for her abdominal symptoms, but no organic cause was found. Her symptoms persisted even after she moved to a new place of residence. A few months later, she began experiencing auditory hallucinations. She had heard 3 unfamiliar male and female voices holding a conversation about her. She had also seen a black shadow in the toilet on one occasion. She was convinced that her third sister had cast black magic on her and sought religious intervention.

However, her symptoms persisted and continued to deteriorate over the next 10 years. She was spending an increasing amount of time sitting on the toilet trying to clear her bowels, due to the constant abdominal discomfort. The sensation of a strong force holding her shoulders down persisted and she remained seated on the toilet watching television, eating her meals, and sleeping. She had lost more than 20 kilograms and was so physically frail from the prolonged immobilization that she was unable to ambulate independently and required assistance to shower. She became easily startled when she heard noises outside her door. She had threatened to kill herself each time her husband persuaded her to seek treatment. She had been sitting continuously on her toilet for 2.5 years before her husband called an ambulance to send her to hospital.

### 2.2. Treatment, Outcome, and Follow-Up

On admission, she was noted to be emaciated and unkempt with hallow cheeks and excoriations over her back and lower limbs. She was unable to ambulate independently, and she had significant wasting of her lower limbs and severe kyphosis. She was also agitated and irritable, expressing multiple somatic complaints. She also verbalized persecutory delusions about her third sister and displayed symptoms of somatic passivity. She had no insight into her mental illness.

Basic blood investigations were done to exclude any organic causes. Full blood count, renal function, electrolytes, liver function, and thyroid function tests were normal except for low vitamin D levels.

She was diagnosed with late-onset schizophrenia and was treated with fluvoxamine 100 milligrams daily, risperidone 4 milligrams daily, and clonazepam 0.5 milligrams daily. She was also started on calcium carbonate and vitamin D tablets daily for her vitamin D deficiency.

In the ward, her persecutory delusions and somatic passivity symptoms reduced with treatment. She did not report any further auditory or visual hallucinations. She spent less than 30 minutes a day in the toilet as the presence of a strong force holding her down reduced. She received intensive physiotherapy but remained wheelchair bound. She was eventually discharged after 18 days in hospital. She continued to follow-up at the outpatient clinic over the next 10 years, and her schizophrenia remained stable with fluvoxamine 100 milligrams daily and a reduction of risperidone to 3 milligrams daily. She continued to have some residual somatic passivity symptoms which she attributed to her mental illness. She developed better insight into her illness and no longer had a fixed firm belief that her third sister was casting black magic on her. She remained compliant to her treatment and follow-up.

## 3. Discussion

From our knowledge, this is the first psychiatric case report of a 57-year-old woman sitting continuously on the toilet for 2.5 years due to her psychotic symptoms. There was a similar news report in 2008 about a 35-year-old woman in Kansas who had also been sitting on her boyfriend's toilet for 2 years before help was sought, but there was no specific mention of the woman's mental health condition [7]. In our patient, her prolonged psychotic symptoms as well as a decline in functioning were in keeping with a diagnosis of schizophrenia rather than a dissociative process triggered by cultural beliefs.

The long DUP in our patient highlights the treatment gap in people with mental illness. A national mental health survey of the Singaporean population in 2009 showed large treatment gaps in those with mental illness. Only 31.7% of people sought help from mental health providers and 8.4% visited her general practitioners while 7.6% sought help from religious healers [8]. A study of mental illness in Asian cultures [9] also showed that there were feelings of fear and resentment amongst relatives of patients with mental health issues within the lower socioeconomic class, while feelings of shame and guilt were common within the higher socioeconomic class.

According to Goffman [10], the perceptions of negative difference (deviance) and their evocation of adverse social responses (stigma) rather than the functional limitations of impairment constitute the greatest problem. Mental health stigma is one of the greatest barriers to seeking help [11]. There is a strong psychiatric stigma attached to the Chinese families due to the burden of intense shame and guilt. This is related to the Chinese culture of fear of exposing its own shame to outsiders. Denial and somatization are often used in relieving stigmatisation.

There is also a lack of mental health literacy amongst Singaporeans. A 2014 study of mental health literacy of 3006 Singapore residents between 18 to 65 years old showed significant stigma towards people with mental illness [12]. In addition, her neighbours' indifference to her lack of well-being may be attributed to the bystander's effect [13].

Her low education levels could also have played a part in her prolonged DUP. Pang et al. [14] reported that people with primary or secondary education were more likely than those with university education to attribute mental health issues to physical causes. The most common symptom manifestation ascribed to Chinese psychiatric patients was psychological distress in the form of bodily symptoms [15]. This could have stemmed from Confucianism values where preservation of social harmony is achieved through emotional avoidance. She had somatic symptoms of incomplete voiding and somatic passivity symptoms of a strong force holding her shoulders down which led to her sitting on the toilet for prolonged periods. Her husband had brought her to see by a general practitioner on several occasions for her abdominal discomfort but had never reported her unusual behaviour to the doctor. The denial of mental illness by her family members could also have caused a delay in seeking psychiatric treatment.

Cultural beliefs also play an important part role in the explanatory model of mental illness [16]. Tsoi [17] reported a special culture form of delusion common in Southeast Asia of being affected by a special type of black magic known as “kongtow.” There is a belief amongst the locals that it is possible to use black magic to cast a spell on them, and females tend to direct their paranoid ideas towards their family members. The delay in seeking help is perpetuated by her family's belief that her unusual behaviour was due to black magic being cast on her.

The phenomena of suffering from black magic tend to be vague and, patients are usually unable to elaborate on it. It is not uncommon for psychiatric patients to suffer symptoms for a prolonged period before seeking help. A review of the psychiatric history of Chinese patients attending a new case clinic showed that more than half of the patients had suffered symptoms for more than a year prior to psychiatric consultation, and 10% had symptoms persisting for more than 9 years [18].

## 4. Conclusion

Psychiatric outreach is easily accessible in the island of Singapore where the total land area is approximately 719.2 square kilometres [19]. There are community mental health services such as the Community Mental Health Team (CMHT) and mobile crisis team, which can be activated by patients or their family members through the mental health helpline. Community partners like family service centres, day activity centres, and police are available to identify and refer similar cases to mental health services. There is a Caregivers Alliance to provide support for caregivers. Despite this, there remains a delay in seeking psychiatric treatment. Indeed, more can be done to improve public awareness and reduce social stigma about mental illness through public educations like mass media, forums, and exhibitions as well as establishing networks with general practitioners, schools, and counselling services.

## Data Availability

Not applicable: all relevant data is contained in the manuscript.
